# A Copy Number Variant on Chromosome 20q13.3 Implicated in Thinness and Severe Obesity

**DOI:** 10.1155/2015/623431

**Published:** 2015-12-31

**Authors:** Sandra J. Hasstedt, Yuanpei Xin, Rong Mao, Tracey Lewis, Ted D. Adams, Steven C. Hunt

**Affiliations:** ^1^Department of Human Genetics, University of Utah School of Medicine, Salt Lake City, UT 84112, USA; ^2^Cardiovascular Genetics Division, University of Utah School of Medicine, Salt Lake City, UT 84112, USA; ^3^Department of Pathology, University of Utah School of Medicine, Salt Lake City, UT 84112, USA; ^4^ARUP Institute for Clinical and Experimental Pathology, Salt Lake City, UT, USA; ^5^Department of Genetic Medicine, Weill Cornell Medical College in Qatar, Doha, Qatar

## Abstract

*Background/Objectives.* To identify copy number variants (CNVs) which are associated with body mass index (BMI).* Subjects/Methods.* CNVs were identified using array comparative genomic hybridization (aCGH) on members of pedigrees ascertained through severely obese (BMI ≥ 35 kg/m^2^) sib pairs (86 pedigrees) and thin (BMI ≤ 23 kg/m^2^) probands (3 pedigrees). Association was inferred through pleiotropy of BMI with CNV log⁡2 intensity ratio.* Results.* A 77-kilobase CNV on chromosome 20q13.3, confirmed by real-time qPCR, exhibited deletions in the obese subjects and duplications in the thin subjects (*P* = 2.2 × 10^−6^). Further support for the presence of a deletion derived from inference by likelihood analysis of null alleles for SNPs residing in the region.* Conclusions.* One or more of 7 genes residing in a chromosome 20q13.3 CNV region appears to influence BMI. The strongest candidate is* ARFRP1*, which affects glucose metabolism in mice.

## 1. Introduction

A number of single nucleotide polymorphisms (SNPs) have been reported to be associated with obesity [1]. Variants in some of the same genes are possibly associated with thinness [2]. Nevertheless, all body mass index- (BMI-) associated SNPs in combination fall short of accounting for the heritability of BMI [1].

Copy number variants (CNVs) may explain additional heritability of BMI. By definition, CNVs are chromosomal regions with sizes of 1 kilobase (kb) to several megabases (Mb) present in variable numbers in different individuals [3]. The Database of Genomic Variants (http://dgv.tcag.ca/dgv/app/home) currently lists >6,000,000 CNVs.

Eighty-four regions have been reported to harbor CNVs that are associated with obesity or BMI [4], including African Americans [5, 6] and Chinese [7, 8]. However, for only 4 of the 84 regions association has been reported in multiple studies [4]. One of the 4 regions, a deletion on chromosome 1p31.1 [9], has also been identified through association with a SNP near* NEGR1* [10, 11]. In 3 other regions, obesity cooccurring with developmental delay was associated with deletions on chromosomes 10q11.22 [7, 9], 16p12.3 [4, 12], and 16p11.2 [13–17]. In addition, an enrichment of CNVs was observed in obese adults [18], severely obese children [19], and children with syndromic obesity [20].

Herein, we performed a genome-wide search for CNVs that associated with BMI in members of extended pedigrees selected either for severe obesity or for healthy thinness, thereby excluding individuals with eating disorders, the focus of most previous genetic studies of the lower end of the BMI range [21]. We studied extended pedigrees to increase our power to detect both common and rare CNVs associated with BMI. To identify associated CNVs, we tested for pleiotropy with BMI, thereby requiring both inheritance and correlation of any identified CNV.

## 2. Subjects and Methods

### 2.1. Subjects

Obese pedigrees were ascertained in Utah using the Health Family Tree, a high school-based family history program designed to teach genetics and disease prevention as part of a mandatory health class [22]. Students, with parental assistance, reported disease information and risk factors for their parents' first-degree relatives. We identified 435 severely obese (BMI ≥ 35 kg/m^2^) sib pairs from the Trees, chose 107 sibships for expansion, and examined 3,333 pedigree members. Similarly, we identified 40 thin (BMI ≤ 23 kg/m^2^) probands who reported multiple thin relatives and examined 400 family members including 265 thin individuals. We also ascertained a set of 1,156 unrelated Roux-en-Y gastric bypass (GB) patients [23]. All subjects were Utah residents with European ancestry. Each project (severe obesity, thinness, and GB) was approved by the Institutional Review Board of the University of Utah and informed consent was obtained.

For every studied member of the pedigrees, height was measured to the nearest centimeter using a Harpenden anthropometer (Holtain, Ltd.). Weight was measured in a hospital gown with a Scaletronix scale (model 5100) (Scaletronix Corporation, Wheaton, IL), which has an 800-pound capacity and weighing accuracy of 0.1 kilograms. BMI was computed as weight divided by height squared.

### 2.2. Laboratory Methods

#### 2.2.1. Array Comparative Genomic Hybridization

CNVs were identified using array comparative genomic hybridization (aCGH) on the NimbleGen platform. A Roche Nimblegen 3x720K genomics array was used for copy number detection. Roche Nimblegen Human CGH3x720K Whole-Genome Tiling array contains empirically tested 720,000 probes per array, which provided analysis of the whole human genome. In brief, genomic DNA was isolated from the leukocytes using QIAamp DNA Blood Kit (Qiagen, Valencia, CA) per the manufacturer's instructions. Microarray analysis was performed following the array manufacturer's protocol. The test and reference genomic DNA were separately labeled with different fluorescent dyes and cohybridized to the 720K whole-genome tiling array. Microarray slides were scanned using Roche Nimblegen MS 200 Microarray Scanner (Roche Nimblegen, Madison, WI) and data captured from the scanned microarray image were saved as TIF files. In the array experiment, each subject was hybridized to the corresponding pedigree control. Data were extracted from scanned images using NimbleScan 2.5 extraction software, which allowed for automated grid alignment, extraction, and generation of data files. Using NimbleScan software, the log⁡2 ratios of the probe signal intensities were calculated. A region was defined as a deletion if there were 5 or more consecutive probes <−0.3. A duplication was defined as 5 or more consecutive probes >0.3. Results on family members were reported as log⁡2 intensity ratio (LRR where R represents intensity) of each CNV compared to a pedigree-specific control. CNVs were also identified for each control and reported as log⁡2 intensity ratio compared to a pooled Promega sample of females (Promega Corp, Madison, WI) in order to eliminate CNVs shared by multiple family members that were also present in the control.

#### 2.2.2. Real-Time qPCR

To validate statistically significant aCGH results, we used real-time qPCR to detect copy number gain or loss, thereby ensuring that the probes in a region were not hybridizing with a homologous region elsewhere in the genome or were false positive findings. Unique sequence primer pairs specific to the targeted regions and spanning regions that are proximal and distal to the breakpoints were designed and used in real-time qPCR reactions using the Duplex TaqMan CNV assay kits (custom TaqMan Copy Number Assay and TaqMan Copy Number Reference Assays which detect a single-copy gene in its respective reference genome assembly; Applied Biosystems, Foster City, CA). The assay consisted of two primers and a FAM-labeled probe. For quality control, each assay was run as a duplex TaqMan real-time PCR reaction, one containing a FAM dye-based assay for the targeted gene and a VIC dye-based assay for the reference gene RNase P. All assays were conducted according to the assay protocol (TaqMan Copy Number Assay protocol, Applied Biosystems, Foster City, CA) in an Optical 384-well plate. In brief, the PCR reaction mixture contained the following: 2x TaqMan Universal PCR Master Mix; 20x Copy Number Assay; 20x Copy Number Reference Assay; DNase-free water; and genomic DNA sample. Four replicates were used for each sample and the control sample was inserted randomly in each batch. PCR was performed in the Applied Biosystems 7900HT Fast Real-Time System. Real-time data was analyzed using CopyCaller software (Applied Biosystems, Foster City, CA). The number of copies of the target sequence in each test sample was determined by relative quantitation (RQ) using the comparative CT method. This method measured the CT difference (delta CT) between target and reference sequences and then compared the delta CT values of test samples to a calibrator sample(s) known to have two copies of the target sequence.

#### 2.2.3. SNP Genotyping

To test for a deletion, which presents as a null allele in SNP genotypes, we used the LightScanner (BioFire Diagnostics, SLC, UT) to genotype 5 SNPs within the chromosome 20q13.3 CNV region.

### 2.3. Statistical Methods

#### 2.3.1. Association Analysis

We tested association with BMI of each autosomal CNV region containing ≥ 30 individuals for which LRR > 0.3 or LRR < −0.3, a restriction adopted to assure sufficient power and protect against false positives. The analysis used LRR as a continuous variable because of the difficulty in, and reduced power of, assigning specific copy number genotypes from aCGH data [24]. LRR was assigned as 0 to all controls and to each subject for whom intensity equaled his/her pedigree control. We separately analyzed each range defined by a distinct start and/or endpoint, selecting the largest positive or smallest negative LRR among an individual's multiple measurements for overlapping regions. Consequently, a single LRR measurement might contribute to multiple CNV regions, making the tests nonindependent.

We detected association as pleiotropy between LRR and BMI, thereby requiring that LRR is both inherited and also genetically correlated with BMI and correspondingly accounting for relatedness within the sample. Using likelihood analysis in jPAP [25, 26], we assumed normality of BMI and LRR with parameters, mean, standard deviation, and heritability of BMI and of LRR, and the genetic correlation (pleiotropy) and environmental correlation between BMI and LRR. *P* values were obtained from a 1 df *χ* ^2^ statistic computed as twice the natural logarithm of the ratio of the maximized likelihood with the genetic correlation estimated to the maximized likelihood with the genetic correlation set equal to zero. To account for multiple testing we made a Bonferroni correction for 3,988 tests, the number of independent tests estimated by eliminating any interval that overlapped with an interval for which more individuals had LRR > 0.3 or LRR < −0.3.

#### 2.3.2. Deletion Analysis

Since a deletion presents as a null allele in SNP data, we tested for a nonzero frequency of a third allele in SNP genotypes. For each SNP, we defined two traits, one for each of the two alleles, and assigned each subject a phenotype for each trait corresponding to presence/absence of the respective allele in his/her genotype. Designating the null allele as 0 and the SNP alleles as 1 and 2, trait 1 penetrance was assigned as 98% for genotypes 0/1, 1/1, and 1/2 and 2% for genotypes 0/2 and 2/2. Correspondingly, trait 2 penetrance was assigned as 98% for genotypes 0/2, 2/2, and 1/2 and 2% for genotype 0/1 and 1/1. We thereby allowed a 2% genotyping error rate and assumed that deletion carriers were genotyped as homozygotes.

Applying a 3-allele bivariate model in jPAP [25, 26] to phenotypes corresponding to genotypes uncleaned of Mendelian errors, we estimated allele frequencies. Significance of the null allele frequency was obtained from a 50 : 50 mixture of 0 and 1 df *χ* ^2^ distributions [27] assumed for twice the natural logarithm of the ratio of the likelihood maximized estimating the null allele frequency to the likelihood maximized with the null allele frequency set to zero. Genotype probabilities were computed using jPAP and mean BMI was obtained for individuals with >90% null allele carrier probability.

## 3. Results

We performed array comparative genomic hybridization (aCGH) on a sample of 882 subjects selected from the pedigrees. The majority of subjects (97%) were members of 86 pedigrees ascertained through severely obese sib pairs; the rest was members of 3 pedigrees ascertained through thin probands (Table 1). For each pedigree, we selected as a control one family member lacking the ascertainment trait: for 18 obese pedigrees, the control was thin, defined in our analysis as a BMI ≤ 23 kg/m^2^; for all other pedigrees, including the 3 thin pedigrees, the control was nonobese (23 kg/m^2^ < BMI ≤ 30 kg/m^2^).

Every subject harbored CNVs defined as LRR > 0.3 or LRR < −0.3; the number of CNVs per subject ranged from 3 to 589 with a mean of 142 (Table 2). Despite wide variation between subjects, the variation in the number of CNVs could not be attributed to either subject's obesity status or pedigree type (Table 2).

We tested for association as pleiotropy between BMI and LRR in 84 CNV regions that were previously reported to be associated with obesity [4]. 37 of the 84 regions contained no CNVs in our sample. For another 36 regions, fewer than 30 subjects harbored CNVs. None of the remaining 11 regions showed association with BMI.

However, BMI showed significant pleiotropy with 3 distinct CNV regions: on chromosomes 11, 18, and 20 (Table 3). Negative genetic correlations estimated for all three regions corresponded to deletions in the obese subjects and duplications in the thin subjects. The significant CNVs ranged from 3 and 12 kb on chromosomes 18 and 11, respectively, to 77 kb on chromosome 20. While the regions on chromosomes 11 and 18 were intergenic, the region on chromosome 20 encompassed 7 genes (*RTEL1*,* TNFRSF6B*,* ARFRP1*,* ZGPAT*,* LIME1*,* SLC2A4RG*, and* ZBTB46*).

To confirm the aCGH-detected CNVs, we applied qPCR to selected subjects from the aCGH sample. For each of the chromosomes 11, 18, and 20 regions, we tested an obese sib pair with aCGH-detected deletions and a thin sib pair with aCGH-detected duplications, plus additional relatives of each sib pair. In the chromosome 20 region, both obese sibs exhibited 1 copy and both thin sibs exhibited 3 copies; furthermore, a third deletion/duplication was found among relatives of the obese/thin sib pair (Table 4).

In contrast, qPCR on chromosomes 11 and 18 identified 2 copies for all tested subjects. This implies that the findings were either off-target effects of the CNV probes or false positive effects from the aCGH methodology.

As further evidence of a deletion on chromosome 20, we genotyped regional SNPs on 3,217 subjects, including the unrelated gastric bypass patients and members of the severe obesity and thin pedigrees, regardless of inclusion within the CNV sample. We obtained significant null allele frequencies for 4 of 5 SNPs in the presumed deletion region (Table 5). The null allele frequencies, ranging from 0.7% to 1.4%, were undoubtedly underestimated since detection of null alleles required that a parent and offspring be homozygous for different alleles. For comparison, significance of a null allele was not obtained for any of 3 SNPs (rs981782, rs1288775, and rs7182723) with similar MAF (0.26 to 0.46) that reside on other chromosomes and were genotyped on the same sample. Null allele carriers for the chromosome 20 SNPs were obese, despite being based on very small numbers (Table 5).

## 4. Discussion

We inferred a BMI-associated CNV on chromosome 20q13.3. Deletions and duplications in the region, identified in our sample using aCGH and confirmed using qPCR, were previously reported for HapMap data with designation esv2758806 [28]. Our inference of null alleles for regional SNPs provided additional evidence for a regional deletion.

Association of BMI with the chromosome 20q13.3 CNV was inferred from pleiotropy, which required that LRR demonstrate inheritance within the families as well as genetic correlation with BMI; we therefore exploited the relatedness within the sample to increase power and protect against false positives. Our qPCR and SNP genotype results also supported association with BMI.

The 77 kb CNV region on chromosome 20q13.3 harbors 7 genes (*RTEL1*,* TNFRSF6B*,* ARFRP1*,* ZGPAT*,* LIME1*,* SLC2A4RG*, and* ZBTB46*). Of these, the best candidate for an effect on BMI is* ARFRP1*, a GTPase that regulates protein trafficking between intracellular organelles. GTPases are involved with signaling of G protein-coupled receptor pathways and were previously implicated for CNVs associated with obesity [19].* ARFRP1* participates in the control of lipid droplet and chylomicron formation [29] and has been shown to affect glucose metabolism in the mouse [30].

The chromosome 20q13.3 CNV exerts a dosage effect on BMI such that hemizygosity results in obesity and duplication results in thinness. A similar dosage effect reported for a CNV on chromosome 16p11.2 differs in the cooccurrence of developmental defects with obesity/thinness [16]. Dosage effects increase the power for CNV detection; the chromosome 20q13.3 CNV was undetectable upon excluding thin sample members and we detected no CNVs that affected exclusively obesity or exclusively thinness.

We limited testing to regions with CNVs detected in a minimum of 30 sample members to assure sufficient power. Subsequent testing of regions with 20–29 CNV-positive individuals using parametric analysis and with 10–19 CNV-positive individuals using nonparametric analysis (results not shown) revealed no additional BMI-associated CNVs. One explanation for the absence of rare CNVs is the corresponding absence in our sample of developmental or other syndromes that have been reported in conjunction with most rare obesity-associated CNVs.

Using linkage analysis on a subset of our obese pedigrees, we previously localized a susceptibility gene to chromosome 4p14, later identified as* TBC1D1* [31], as well as yet unidentified susceptibility genes on chromosomes 4q34-35 and 20q13.1 [32]. The chromosome 20 linkage region falls 20 MB centromeric of the CNV detected herein and no CNV was detected on chromosome 4q; therefore, none of our reported linkage regions can be attributed to a CNV. Chromosome 20q13 has been identified through linkage analysis of BMI in other samples as well [33–36] but is always more centromeric than the CNV reported herein.

Despite the inference of numerous obesity susceptibility regions across the genome, all confirmed variants in combination fail to fully account for the heritability of BMI [37]. Obesity gene discovery is complicated by complex inheritance that includes genetic and environmental interactions [38]. Although the contribution of the chromosome 20q13.3 CNV to inherited obesity awaits confirmation, the region contains a strong candidate in* ARFRP1*. In addition, future obesity investigations should consider a strength of our study: the increased power provided by the inclusion of thin subjects.

In summary, we detected a CNV on chromosome 20q13.3 that is hemizygous in the obese subjects and duplicated in the thin subjects. The region encompasses 7 genes of which* ARFRP1* appears to be the best candidate.

## Figures and Tables

**Table 1:** Counts of subjects and family controls in the aCGH sample by pedigree type and BMI range.

Pedigree type	Subjects				Family controls		
	BMI ≤ 23	23 < BMI < 35	BMI ≥ 35	Total	BMI ≤ 23	23 < BMI < 30	Total
Obese	6	76	685	767	30	56	86
Thin	26	0	0	26	0	3	3
Total	32	76	685	793	30	59	89

**Table 2:** Mean, minimum, and maximum number of CNVs (LRR > 0.3 or LRR < −0.3) per subject by BMI range and by pedigree type.

CNV Count per subject	BMI range			Pedigree type	
	BMI ≤ 23	23 < BMI < 35	BMI ≥ 35	Thin	Obese
Mean	151	150	141	105	141
Minimum	17	27	3	50	50
Maximum	424	485	589	168	435

**Table 3:** CNV regions attaining significant pleiotropy with BMI.

Chromosome	Position (bp)^1^		CNV heritability	Genetic correlation	Nominal *P*
	Start	End			
11	65016627	65028410	0.312	−0.978	0.00000556
18	33143732	33146462	0.335	−0.899	0.00000112
20	62303103	62380372	0.198	−1.000	0.00000215

^1^Build GRCh37.

**Table 4:** Copy number of *SLC2A4RG* on chromosome 20 measured by qPCR on selected members of one thin and one obese pedigree. Replicate qPCR measurements on four individuals fell within 3% of the original measurement and qPCR on a local pooled female sample exhibited 2 copies.

Pedigree type	Relationship^1^	Gender	Age	BMI	LRR	Copy number by qPCR^2^
Obese	Index	F	38	41.9	−0.5277	1
Obese	Sibling	F	36	38.8	−0.6893	1
Obese	Sibling	F	38	28.0	0.0000	2
Obese	Mother	F	56	48.5	0.2475	2
Obese	Grandfather	M	88	27.8	−0.0659	2
Obese	Aunt	M	53	37.1	−0.1190	2
Obese	Aunt	F	48	61.0	0.3003	2
Obese	Aunt	F	43	43.1	−0.4856	1
Obese	Cousin	F	22	43.3	0.0279	2
Thin	Index	F	37	19.7	0.3285	3
Thin	Sibling	F	34	20.9	0.3230	3
Thin	Mother	F	65	30.6	NA	2
Thin	Uncle	M	59	20.0	0.2821	2
Thin	Cousin	F	31	18.1	−0.2417	2
Thin	Uncle	M	61	20.8	0.4393	2
Thin	Cousin	F	36	17.3	0.0810	2
Thin	Cousin	F	NA	NA	NA	3

^1^Relationship to the index case in each pedigree through her mother.

^2^Promega female sample as standard.

**Table 5:** Chromosome 20 SNPs tested for evidence of null alleles.

SNP				Null allele			
rs#	Position (bp)^1^	Gene	MAF^2^	Frequency	*P* value	*N* ^3^	BMI
rs6062302	62320968	*RTEL1*	0.235	0.00775	0.002222	6	33.9
rs2257440	62328267	*TNFRSF6B*	0.226	0.00724	0.008077	2	32.8
rs6062498	62339059	*ZGPAT*	0.328	0.01377	0.005117	0	—
rs6062510	62372148	*SLC2A4RG*	0.308	0.00000	1.000000	0	—
rs932824	62380315	*ZBTB46*	0.209	0.01274	0.000181	3	30.2

^1^Build GRCh37.

^2^Minor allele frequency estimated from our sample.

^3^Number of deletion carriers with genotype probability > 90%.

## References

[1] Sandholt C. H., Hansen T., Pedersen O. (2012). Beyond the fourth wave of genome-wide obesity association studies. *Nutrition and Diabetes*.

[2] Zegers D., Beckers S., Hendrickx R. (2013). Prevalence of rare MC3R variants in obese cases and lean controls. *Endocrine*.

[3] Conrad D. F., Pinto D., Redon R. (2010). Origins and functional impact of copy number variation in the human genome. *Nature*.

[4] Peterson R. E., Maes H. H., Lin P. (2014). On the association of common and rare genetic variation influencing body mass index: a combined SNP and CNV analysis. *BMC Genomics*.

[5] Zhao W., Wineinger N. E., Tiwari H. K. (2012). Copy number variations associated with obesity-related traits in African Americans: a joint analysis between GENOA and HyperGEN. *Obesity*.

[6] Yang T.-L., Guo Y., Shen H. (2013). Copy number variation on chromosome 10q26.3 for obesity identified by a genome-wide study. *The Journal of Clinical Endocrinology & Metabolism*.

[7] Sha B.-Y., Yang T.-L., Zhao L.-J. (2009). Genome-wide association study suggested copy number variation may be associated with body mass index in the Chinese population. *Journal of Human Genetics*.

[8] Sun C., Cao M., Shi J. (2013). Copy number variations of obesity relevant loci associated with body mass index in young Chinese. *Gene*.

[9] Jarick I., Vogel C. I. G., Scherag S. (2011). Novel common copy number variation for early onset extreme obesity on chromosome 11q11 identified by a genome-wide analysis. *Human Molecular Genetics*.

[10] Willer C. J., Speliotes E. K., Loos R. J. (2009). Six new loci associated with body mass index highlight a neuronal influence on body weight regulation. *Nature Genetics*.

[11] Speliotes E. K., Willer C. J., Berndt S. I. (2010). Association analyses of 249,796 individuals reveal 18 new loci associated with body mass index. *Nature Genetics*.

[12] Yang T.-L., Guo Y., Li S. M. (2013). Ethnic differentiation of copy number variation on chromosome 16p12.3 for association with obesity phenotypes in European and Chinese populations. *International Journal of Obesity*.

[13] Walters R. G., Jacquemont S., Valsesia A. (2010). A new highly penetrant form of obesity due to deletions on chromosome 16p11.2. *Nature*.

[14] Bachmann-Gagescu R., Mefford H. C., Cowan C. (2010). Recurrent 200-kb deletions of 16p11.2 that include the SH2B1 gene are associated with developmental delay and obesity. *Genetics in Medicine*.

[15] Bochukova E. G., Huang N., Keogh J. (2010). Large, rare chromosomal deletions associated with severe early-onset obesity. *Nature*.

[16] Jacquemont S., Reymond A., Zufferey F. (2011). Mirror extreme BMI phenotypes associated with gene dosage at the chromosome 16p11.2 locus. *Nature*.

[17] Walters R. G., Coin L. J., Ruokonen A. (2013). Rare genomic structural variants in complex disease: lessons from the replication of associations with obesity. *PLoS ONE*.

[18] Wang K., Li W.-D., Glessner J. T., Grant S. F. A., Hakonarson H., Price R. A. (2010). Large copy-number variations are enriched in cases with moderate to extreme obesity. *Diabetes*.

[19] Wheeler E., Huang N., Bochukova E. G. (2013). Genome-wide SNP and CNV analysis identifies common and low-frequency variants associated with severe early-onset obesity. *Nature Genetics*.

[20] Vuillaume M.-L., Naudion S., Banneau G. (2014). New candidate loci identified by array-CGH in a cohort of 100 children presenting with syndromic obesity. *American Journal of Medical Genetics A*.

[21] Helder S. G., Collier D. A. (2011). The genetics of eating disorders. *Behavioral Neurobiology of Eating Disorders*.

[22] Williams R. R., Hunt S. C., Barlow G. K. (1988). Health family trees: a tool for finding and helping young family members of coronary and cancer prone pedigrees in Texas and Utah. *American Journal of Public Health*.

[23] Adams T. D., Avelar E., Cloward T. (2005). Design and rationale of the Utah obesity study. A study to assess morbidity following gastric bypass surgery. *Contemporary Clinical Trials*.

[24] Marenne G., Real F. X., Rothman N. (2012). Genome-wide CNV analysis replicates the association between GSTM1 deletion and bladder cancer: a support for using continuous measurement from SNP-array data. *BMC Genomics*.

[25] Hasstedt S. J. (2005). jPAP: document-driven software for genetic analysis. *Genetic Epidemiology*.

[26] Hasstedt S. J., Thomas A. (2011). Detecting pleiotropy and epistasis using variance components linkage analysis in jPAP. *Human Heredity*.

[27] Self S. G., Liang K.-Y. (1987). Asymptotic properties of maximum likelihood estimators and likelihood ratio tests under nonstandard conditions. *Journal of the American Statistical Association*.

[28] Redon R., Ishikawa S., Fitch K. R. (2006). Global variation in copy number in the human genome. *Nature*.

[29] Hesse D., Jaschke A., Chung B., Schürmann A. (2013). Trans-Golgi proteins participate in the control of lipid droplet and chylomicron formation. *Bioscience Reports*.

[30] Hesse D., Jaschke A., Kanzleiter T. (2012). GTPase ARFRP1 is essential for normal hepatic glycogen storage and insulin-like growth factor 1 secretion. *Molecular and Cellular Biology*.

[31] Stone S., Abkevich V., Russell D. L. (2006). TBC1D1 is a candidate for a severe obesity gene and evidence for a gene/ gene interaction in obesity predisposition. *Human Molecular Genetics*.

[32] Hunt S. C., Abkevich V., Hensel C. H. (2001). Linkage of body mass index to chromosome 20 in Utah pedigrees. *Human Genetics*.

[33] Borecki I. B., Rice T., Pérusse L., Bouchard C., Rao D. C. (1994). An exploratory investigation of genetic linkage with body composition and fatness phenotypes: the Québec Family Study. *Obesity Research*.

[34] Lee J. H., Reed D. R., Li W. D., Lee J. H. (1999). Genome scan for human obesity and linkage to markers in 20q13. *The American Journal of Human Genetics*.

[35] Shmulewitz D., Heath S. C., Blundell M. L. (2006). Linkage analysis of quantitative traits for obesity, diabetes, hypertension, and dyslipidemia on the island of Kosrae, Federated States of Micronesia. *Proceedings of the National Academy of Sciences of the United States of America*.

[36] Kettunen J., Perola M., Martin N. G. (2009). Multicenter dizygotic twin cohort study confirms two linkage susceptibility loci for body mass index at 3q29 and 7q36 and identifies three further potential novel loci. *International Journal of Obesity*.

[37] Lee Y. S. (2013). Genetics of nonsyndromic obesity. *Current Opinion in Pediatrics*.

[38] Albuquerque D., Stice E., Rodríguez-López R., Manco L., Nóbrega C. (2015). Current review of genetics of human obesity: from molecular mechanisms to an evolutionary perspective. *Molecular Genetics and Genomics*.

